# The Treatment of Giant Periurethral Condyloma in Pregnancy Using an Ultrasonic Thermal Scalpel: A Case Report and New Single Session Treatment Option

**DOI:** 10.1155/2015/792412

**Published:** 2015-01-12

**Authors:** Ali Yavuzcan, Mete Çağlar, Hakan Turan, Ali Tekin, Seren Topuz, Gizem Yavuzcan, Serdar Dilbaz, Yusuf Üstün, Cihangir Aliağaoğlu, Selahattin Kumru

**Affiliations:** ^1^Department of Obstetrics & Gynaecology, Düzce University Faculty of Medicine, 81000 Düzce, Turkey; ^2^Department of Dermatology, Düzce University Faculty of Medicine, 81000 Düzce, Turkey; ^3^Department of Urology, Düzce University Faculty of Medicine, 81000 Düzce, Turkey

## Abstract

Multiple large polypoid lesions with exophytic appearance occurring in anal and perineal region as a result of human papilloma virus (HPV) infection are referred to as giant condyloma acuminatum (GCA). The conventional treatment of these lesions involves the use of surgical excision, laser, electrocautery, and/or application of trichloroacetic acid. A 28-year-old primigravid patient at 22 weeks of pregnancy presented to the hospital complaining of vaginal bleeding and palpable mass in the vulva. The physical examination revealed a 60 × 35 mm broad-based, fragile, and patchy hemorrhagic polypoid lesion originating 1 cm below the clitoris and completely occupying urethral orifice and partially occluding vaginal vestibule. The patient underwent excision of GCA in the midtrimester using an ultrasonic thermal scalpel (Harmonic Scalpel) without any additional treatment and subsequently delivered a single live healthy baby. The excision of GCA occurring during pregnancy using Harmonic Scalpel can be regarded as a new successful method. Prospective, randomized, and controlled studies are warranted in order to provide clear evidence of the efficiency and safety of HS in the treatment of GCA.

## 1. Introduction

Multiple large polypoid lesions with exophytic appearance occurring in anal and perineal region as a result of human papilloma virus (HPV) infection are referred to as Buschke-Löwenstein tumor (BLT) or giant condyloma acuminatum (GCA) [1]. The prevalence of HPV infection is around 46% during pregnancy. The condylomas tend to enlarge and increase in number during pregnancy due to physiological changes in the external genital organs and partial suppression in the immune system [2, 3]. These tumor-like structures exhibit a very low potential of malignant degeneration, and the conventional treatment of these lesions involves the use of surgical excision, laser, electrocautery, and/or application of trichloroacetic acid. The rate of recurrence is high following these therapies. More than one session is usually needed for a successful treatment. Furthermore, conventional therapies are also associated with some complications such as severe hemorrhage, local bacterial infections, pain, preterm delivery, and abortion [4].

The ultrasonic thermal scalpel (Harmonic Scalpel) is an ultrasonic device that simultaneously performs cutting and coagulation using ultrasonic vibration. The device offers the advantages of causing minimal lateral thermal damage and producing lesser smoke. In addition, the device avoids the passage of electrical current and neuromuscular stimulation [5]. To our knowledge, the Harmonic Scalpel (HS) has not been previously used for the treatment of a giant periurethral condyloma in the presence of a healthy intrauterine pregnancy.

We reported a patient who underwent excision of GCA in the midtrimester using a HS and who subsequently delivered a single live baby.

## 2. Case

**Figure 1:**
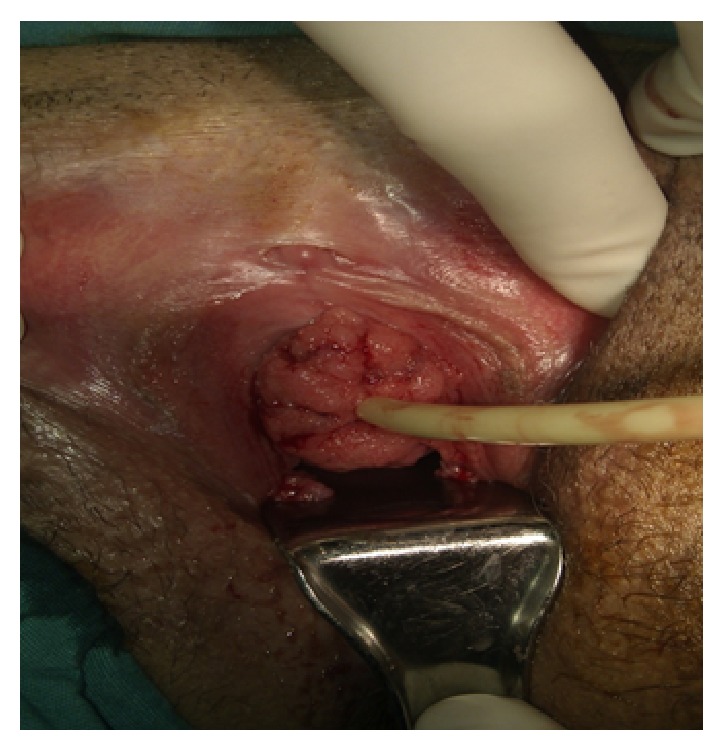
GCA was detected that completely surrounded the external urethral orifice.

A 28-year-old primigravid patient at 22 weeks of pregnancy presented to the hospital complaining of vaginal bleeding and palpable mass in the vulva. The physical examination revealed a 60 × 35 mm broad-based, fragile, and patchy hemorrhagic polypoid lesion originating 1 cm below the clitoris and completely occupying urethral orifice and partially occluding vaginal vestibule (Figure 1). In addition, polypoid pedunculated lesions were observed, one in the posterior fourchette and one between the labia minora. The cervicovaginal examination of the patient did not reveal any pathological lesion in the cervix or on the vaginal wall.

On ultrasonography (USG), biometric measurements were consistent with a single live fetus with a gestational age of 22 weeks and 2 days. The routine hematological and biochemical tests did not reveal any pathological findings. The tests performed for syphilis, human immune deficiency virus (HIV), and hepatitis virus subtypes were found to be negative. Polyacrylamide gel electrophoresis and multiplex polymerase chain reaction (PCR) performed in cervicovaginal samples revealed HPV type 6. The urological examination performed in the partner of the patient did not reveal any finding.

**Figure 2:**
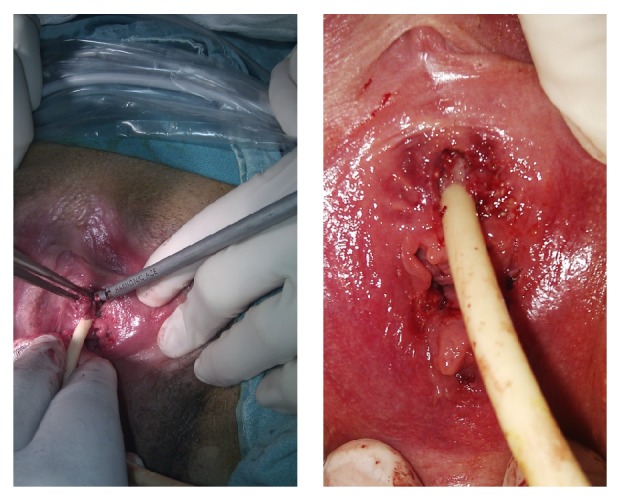
The excision of GCA using HS and appearance after the operation.

The decision to perform condyloma excision under spinal anesthesia was made. The patient was provided detailed explanation about the procedure, and a signed written informed consent was obtained. The pedunculated condylomas in the labia minora and GCA in periurethral region were excised using HS. The HS (The Harmonic ACE, Ethicon Endo-Surgery Inc., Cincinnati, OH, USA) was used according to the manufacturer's instructions. It has a shaft diameter of 5 mm and length of 36 mm. The device has different power levels for different tissues. HS was operated at power level 3 for GCA. Duration of the application of ultrasonic energy did not take more than 10 seconds for each lesion. The lesions were dissected and superficial epidermal destruction was done without any cavitation (Figure 2).

The procedure was performed using a cystoscopy with the assumption that condylomas could also be present in the urethra neck and bladder. There was no pathological finding in the ureter orifices and urethra. The pathological examination was consistent with condyloma acuminatum. No surgical or obstetric complications occurred in the postoperative period. The patient continued her routine pregnancy follow-up. The patient did not develop recurrent or new condyloma until delivery. The patient delivered a 2770-gram male baby with cesarean section due to fetal distress at gestational age of 36 weeks and 6 days. The patient did not have any symptoms at 12 months postpartum.

## 3. Discussion

Active vulvovaginal HPV type 6 and 11 infections may exhibit variable clinical courses. The virus can be eliminated spontaneously, enter into a latent phase, cause subclinical infection for long years, produce benign lesions, and turn into a precancerous lesion with accompanying oncogenic subtypes or result in tumor-like masses induced by HPV [6]. The tumor-like masses induced by HPV infection whose sizes range between few mm and 1-2 cm are called condyloma acuminatum (CA). In nonpregnant women, 30% of CA show spontaneous regression under the influence of humoral and cellular immunity. Months and even years after recovery of the visible lesions, viral genome can be detected in the normal epithelium (latent or subclinical infection) [7]. GCA do not exhibit spontaneous regression during pregnancy, and recurrence is common due to treatment failure [8]. Maternal complications such as vaginal bleeding, vaginal obstruction, and urethral obstruction can occur that may result in an increased rate of cesarean section [9–11]. On the other hand, Cohen et al. did not report an increased rate of complications in fetuses born to mothers with CA [11]. The current patient was admitted to the hospital complaining of vaginal bleeding and palpable mass. The cervicovaginal examination of the patient did not reveal any soft issue lesion that would prevent normal vaginal delivery; however, a GCA was detected that completely surrounded the external urethral orifice (Figure 1).

The treatment choice for GCA is very important during pregnancy. No definitive evidence suggests that any of the available treatments are superior to any other, and no single treatment is ideal for all patients or all warts. The use of locally developed and monitored treatment algorithms has been associated with improved clinical outcomes and should be encouraged [12]. Trichloroacetic acid, liquid nitrogen, laser ablation, or electrocautery can be used to treat external genital HPV lesions at any time during pregnancy [13]. Imiquimod, sinecatechins, podophyllin, and podofilox should not be used freely during pregnancy [12].

Rarely, HPV types 6 and 11 can cause respiratory papillomatosis in infants and children, although the route of transmission (i.e., transplacental, perinatal, or postnatal) is not completely understood [12]. Cesarean delivery may be indicated for women with genital warts if the pelvic outlet is obstructed or if vaginal delivery would result in excessive bleeding [12]. Pregnant women with genital warts should be counseled concerning the low risk for warts on the larynx (recurrent respiratory papillomatosis) in their infants or children [12]. The surgery is the most commonly preferred treatment modality, and it has proved efficiency in the early stages of the disease [8]. The surgical excision can be performed using classical surgical techniques or using electrocautery. The large tissue defect occurring in the vulvar region after the excision of a giant tumor may increase the rate of complications such as insufficient tissue healing, inflammation and infection, abortion, and preterm delivery. It is therefore recommended to perform partial-thickness skin grafting after excision of GCA [14]. In the present case, primary surgical excision and subsequent repair was considered in the first place. However, an alternative method has been planned due to the risk of urinary infection and permanent urethral stenosis following radical excision in periurethral area and tissue grafting.

Chu et al. reported recurrence in 50% of nonpregnant women with anogenital GCA that underwent radical surgery using traditional methods [15]. This has led to the consideration of the use of adjuvant therapies in order to increase the success rate of therapies in GCA. Safi et al. reported that cryotherapy followed by topical application of podophyllin 25–30% or trichloroacetic acid 25% remained insuffıcient in 25% of the nonpregnant patients [16]. Interferon therapy or intralesional or systemic chemotherapy can be administered in nonpregnant women before excision. Likewise, radiotherapy increases the success rate of the therapy in nonpregnant women [16]. Erkek et al. reported successful use of surgical excision in conjunction with peroral retinoic acid and topical administration of imiquimod in nonpregnant patients with GCA [17]. As mentioned before, there is a debate over the use of these therapies during pregnancy due to their teratogenic potential. Laser is usually preferred when multiple warts are spread over a large area. Traditional surgical excision would be difficult for treating cervical and vaginal condylomas and CO_2_ laser is a useful option for this aim [18]. There are some presented reports about treatment of GCA using neodymium-YAG or carbon dioxide laser [19]. CO_2_ laser surgery is currently the only treatment option that has proven efficiency in the treatment of complications related to HPV infection during pregnancy. The rate of recurrence during pregnancy or in the postpartum period is extremely low after excision using CO_2_ laser [20]. We could not use CO_2_ laser as we do not have laser device in our center.

HS is commonly employed in laparoscopic and open surgery. HS converts ultrasonic waves into high-frequency mechanical energy, and it simultaneously performs cutting and coagulation in the tissue. The cut surfaces become denaturized and turn into a coagulum which prevents blood loss. The temperature is between 50 and 100°C in procedures performed with HS, and the risk of injury to the surrounding tissues is minimal [21]. A major advantage of ultrasonic scissors is effective vessel sealing, as well as lymphatics [22]. Sealing of lymphatics also may be preventive for dissemination of infective diseases. On the other hand, it was reported that maximum temperatures of about 200°C or higher at the dissecting end of HS maybe occur after activation for 10 seconds [23]. This entails a certain lateral thermal damage and potential injury to adjacent organs [24]. The cutaneous condylomas usually originate from the papillary dermis. The papillary dermis is about 250–300 *μ*m in depth, under the epidermis. There is no well-defined papillary dermis on the vulva [25]. It is known that only superficial destruction of vulvar lesions with laser or traditional electrocoagulation provides successful treatment [26]. We excised the lesion with superficial destruction of vulvar epithelium located at the basement of the lesions. We did not form a cavitation. The HS has different types of devices like Harmonic ACE + Shears, Harmonic Focus Long Curved Shears, Harmonic Wave Open Shears, and so forth [22]. But we had a classical type Harmonic ACE in our center. The other types of these devices may be more useful for treatment of giant condylomas.

The excision of GCA using HS and without using adjuvant therapy is a new treatment method. HS offers the advantages of easy applicability and short procedure time in the excision of GCA. The risk of blood loss, edema, and wound site infection is lower with HS compared to conventional scalpel method [18]. Furthermore, GCA is more commonly encountered in patients with sexually transmitted diseases or HIV. As indicated by Duus et al., HS offers a safer method compared to conventional techniques while performing a procedure in body areas of HIV positive patients that pose a risk of disease transmission for the surgeon [18]. It is considered that the advantages of HS aforementioned even become more important during surgeries performed in pregnant women. The present case also did not develop perioperative or postoperative surgical complications after the excision of GCA using HS. The patient also did not develop recurrence during pregnancy and up to 12 months after delivery.

The excision of GCA occurring during pregnancy using HS can be regarded as a new successful method. Prospective, randomized, and controlled studies are warranted in order to provide clear evidence of the efficiency and safety of HS in the treatment of GCA.
