# Corrigendum to “The Treatment of Giant Periurethral Condyloma in Pregnancy Using an Ultrasonic Thermal Scalpel: A Case Report and New Single Session Treatment Option”

**DOI:** 10.1155/2015/170978

**Published:** 2015-11-02

**Authors:** Ali Yavuzcan, Mete Çağlar, Hakan Turan, Ali Tekin, Gizem Yavuzcan, Seren Topuz, Serdar Dilbaz, Yusuf Üstün, Cihangir Aliağaoğlu, Selahattin Kumru

**Affiliations:** ^1^Department of Obstetrics & Gynaecology, Düzce University Faculty of Medicine, 81000 Düzce, Turkey; ^2^Department of Dermatology, Düzce University Faculty of Medicine, 81000 Düzce, Turkey; ^3^Department of Urology, Düzce University Faculty of Medicine, 81000 Düzce, Turkey

In the paper “The Treatment of Giant Periurethral Condyloma in Pregnancy Using an Ultrasonic Thermal Scalpel: A Case Report and New Single Session Treatment Option” [1], the authors' order is corrected as above.
